# Association of Myopia in Elementary School Students in Jiaojiang District, Taizhou City, China

**DOI:** 10.1155/2021/3504538

**Published:** 2021-02-27

**Authors:** Xin Lu, Congcong Guo, Bin Xu, Chenwei Hou, Xiaoming Huang, Hui Xu, Zhichun Weng

**Affiliations:** ^1^Jiaojiang Center for Disease Control and Prevention, Taizhou, Zhejiang Province, China; ^2^Eye Hospital (Taizhou Branch), WMU International Eye Hospital of Taizhou, Taizhou, Zhejiang Province, China; ^3^Jiaojiang Education Bureau, Taizhou, Zhejiang Province, China

## Abstract

**Background:**

The aim of our study was to evaluate the prevalence of myopia in elementary school students and to assess the risk factors for myopia.

**Methods:**

This school-based cross-sectional study was performed on students from two elementary schools in Jiaojiang, Taizhou City, China. A total of 556 students, whose age ranged from 9 to 12 years, were included. The uncorrected visual acuity and noncycloplegic refractive error tests were performed to determine the myopia. Each student was asked to fulfill the questionnaire about the possible factors associated with myopia. Multivariate logistic analyses of risk factors were conducted.

**Results:**

The overall prevalence of myopia among those students was 63.7%, ranged from 53.4% in grade 4 to 72.5% in grade 6. Multivariate logistic analysis showed that adjusting the height of desks and chairs according to the changing height and the presence of myopia in parents were significantly associated with myopia in these students, respectively.

**Conclusions:**

Our results showed that myopia among elementary school students was associated with environmental and hereditary factors.

## 1. Introduction

Myopia, also known as short-sightedness, is one of the leading causes of visual disability that develops primarily during childhood when excessive elongation of the eyes results in blurry distance vision and clear close vision [1]. The increasing prevalence of myopia is a global health and social problem [2]. Researchers have estimated that about 50% of the world's population will be myopic and about 10% will be high myopic by 2050 [3]. The “myopia boom” is particularly prominent in urban areas of East and Southeast Asia, where 80% to 90% of high school graduates have myopia and about 20% have high myopia [4, 5]. As the most common visual impairment in children, myopia poses an enormous personal and social burden [6]. Additionally, children with high myopia are at high risk of developing irreversible visual impairment or blindness mostly due to retinal detachment, glaucoma, and myopic macular degeneration [7, 8].

Most myopic individuals are associated with excessive axial elongation, and very few occur as a result of disproportionately high corneal power [9]. For adults aged 50 or older, myopia can also be rarely caused by nuclear cataracts [9]. Both environmental and genetic factors impose a significant risk of myopia [10]. The identified genetic variants could explain about 12% of the variance of the refractive error trait [11, 12]. Tideman et al. found that different genetic loci were associated with different ages of axial length (AL) and corneal radius (CR) ratio [13]. Among those younger than 10 years, three loci (GJD2, CHRNG, and ZIC2) were associated with AL/CR. In people aged 10 to 25 years, four loci (BMP2, KCNQ5, A2BP1, and CACNA1D) were associated; and in adults (>25 years of age), 20 loci were associated. Environmental factors such as high levels of education, lack of outdoor exposure, and excessive near-work activities are the most established risk factors for myopia [1, 5]. A Mendelian randomization study by Mountjoy et al. also showed that more time in education may be a causal risk factor for myopia [14]. Since refractive error correction could not prevent the myopic pathologies, preventing the myopia and particularly high myopia at the early age is of great significance [1, 4]. Each year of delay in the age at onset could substantially reduce the chance of developing high myopia in adulthood [15]. With the aim of discovering potentially effective prevention methods during childhood, in this cross-sectional study, we collected children in elementary schools to evaluate the prevalence of myopia in these young populations and assess the protective and risk factors for myopia.

## 2. Materials and Methods

Two elementary schools (school A and school B) in Jiaojiang District, Taizhou City, Zhejiang Province, China, were included. Students from grades 4 to 6 were enrolled from September to October 2019. Two or three classes were randomly selected in each grade, and all students in selected classes were enrolled.

Each participant was asked to fulfill the customized questionnaire, including the characteristics of students and possible factors associated with myopia. The uncorrected visual acuity (UCVA) and noncycloplegic refractive error tests were performed by pediatric ophthalmologists from Taizhou Municipal Hospital. The UCVA was tested using the Standard Logarithmic Visual Acuity E Chart, and noncycloplegic refractive error was tested using the RM-800 Auto Refractometer (Topcon Medical Systems, Inc). The UCVA less than 5.0 and spherical equivalent refraction less than −0.50 diopter in at least one eye were used to define the myopia.

Statistical analyses were conducted using the Statistical Package for the Social Sciences (SPSS, version 21.0; IBM, Chicago). The chi-squared tests were used to evaluate the associations between factors and myopia. The parameters with a univariate association were selected as candidate variates for multivariate logistic analysis. The odds ratio (OR) and 95% confidence intervals (CIs) were calculated. A *P* value of less than 0.05 was considered statistically significant.

## 3. Results

A total of 556 students (310 in school A and 246 in school B) were included in this study. The prevalence of myopia was 63.7%, with 64.8% in school A and 62.2% in school B. There is no statistical difference in the prevalence of myopia between the two schools (*P*=0.520). The prevalence of myopia showed statistically different among grade 4, grade 5, and grade 6 in school B (*P* < 0.001) and total (*P*=0.001), respectively. The average age of students with myopia was higher than those of normal students in school B and total (both *P* < 0.001). No statistical difference in the proportion of myopia was found between males and females (Table 1).

Table 2 shows the associations between factors studied and the prevalence of myopia in primary school students. The frequencies of changing class seats and adjusting the height of desks and chairs were statistically associated with the presence of myopia (*P* < 0.05). Sleeping time more than 8 h and the presence of myopia in parents were also found to be associated with the prevalence of myopia (*P* < 0.05). No other factor showed a univariate association.

After adjusting the age and gender, adjusting the height of desks and chairs according to the changing height and the presence of myopia in parents were still associated with the presence of myopia (all *P* < 0.05, Table 3). Comparing with never adjusting the height of desks and chairs, adjusting the height of desks and chairs once a year and once a semester in total (OR = 0.37, 95% CI = 0.21–0.67, *P*=0.001; OR = 0.60, 95% CI = 0.35–0.97, *P*=0.037) and adjusting the height of desks and chairs once a year in school B (OR = 0.26, 95% CI = 0.11–0.62, *P*=0.003) were protective factors. Parents having no myopia was a protective factor for myopia in total (OR = 0.51, 95% CI = 0.35–0.74, *P* < 0.001), school A (OR = 0.56, 95% CI = 0.34–0.93, *P*=0.026), and school B (OR = 0.45, 95% CI = 0.25–0.83, *P*=0.009), respectively.

## 4. Discussion

In this study, we identified that adjusting the height of desks and chairs according to the changing height and the presence of myopia in parents were associated with myopia in elementary school students.

The prevalence of myopia in our study was 63.7%, which was similar to the myopia prevalence of 66.5% among students of grades 4 to 6 in Yiwu, a county-level city of Zhejiang Province, China [16]. The prevalence of myopia was found to be positively associated with grade and age. For the intervention of myopia, spectacles and contact lenses are considered as the mainstay to improve distance vision [9]. Pharmacological intervention includes nonselective muscarinic antagonist atropine and the M1 receptor-specific antagonist pirenzepine, which are also used to control myopia [17, 18], whereas refractive surgeries including keratorefractive procedures and intraocular procedures are used to correct refractive error [19–21]. Although the symptoms of myopia can be alleviated with those management practices, the risk of complications from potentially blinding conditions such as retinal detachments increase with the longer AL associated with high myopia [7, 22]. The prevention or delay of myopia by controlling environmental and genetic risk factors at the early age should be the priority for myopia control.

Parents having no myopia were identified to be a protective factor for myopia, suggesting hereditary factors may play an important role in myopia. Verhoeven et al. had identified multiple susceptibility loci for refractive error and myopia [11]. Multiple studies have suggested the family history of myopia was significantly associated with myopia [23, 24]. In our study, adjusting the height of desks and chairs according to the changing height was also shown to be a protective factor, possibly due to the rapid change of stature in students of this age. The prevalence and the associations should be interpreted with caution because of the several limitations in this study. First, because of the relatively small sample size, some variates may not show a significant difference between myopic students and normal students, such as outdoor activities. Second, recall bias could exist in this cross-sectional study; hence, a longitudinal cohort trial was needed to further confirm the associations. Third, only two primary schools were included in this study, which led to a selection bias.

## 5. Conclusions

Our results showed that the prevalence of myopia among elementary school students was associated with environmental and hereditary factors.

## Figures and Tables

**Table 1:** Characteristics of students in two elementary schools.

Parameters	All (*n* = 556)			School A (*n* = 310)			School B (*n* = 246)		
	Myopia (*n* = 354)	Normal (*n* = 202)	*P* value	Myopia (*n* = 201)	Normal (*n* = 109)	*P* value	Myopia (*n* = 153)	Normal (*n* = 93)	*P* value
*Age, mean* *±* *SD, y*	10.21 ± 0.89	9.89 ± 0.87	<0.001	10.12 ± 0.89	9.95 ± 0.84	0.112	10.33 ± 0.87	9.82 ± 0.90	<0.001

*Grade, n (%)*									
4	102 (53.4%)	89 (46.6%)	0.001	68 (63.0%)	40 (37.0%)	0.169	34 (41.0%)	49 (59.0%)	<0.001
5	120 (65.6%)	63 (34.4%)		61 (59.8%)	41 (40.2%)		59 (72.8%)	22 (27.2%)	
6	132 (72.5%)	50 (27.5%)		72 (72.0%)	28 (28.0%)		60 (73.2%)	22 (26.8%)	

*Gender*, *n* (%)									
Male	194 (63.2%)	113 (36.8%)	0.795	110 (63.6%)	63 (36.4%)	0.603	84 (62.7%)	50 (37.3%)	0.862
Female	160 (64.3%)	89 (35.7%)		91 (66.4%)	46 (33.6%)		69 (61.6%)	43 (38.4%)	

**Table 2:** The associations between factors and the prevalence of myopia.

Parameters	All (*n* = 556)			School A (*n* = 310)			School B (*n* = 246)		
	Myopia (*n* = 354)	Normal (*n* = 202)	*P* value	Myopia (*n* = 201)	Normal (*n* = 109)	*P* value	Myopia (*n* = 153)	Normal (*n* = 93)	*P* value
*Change class seats, n (%)*									
Never	0 (0.0%)	5 (100.0%)	0.030	0 (0.0%)	3 (100.0%)	0.115	0 (0.0%)	2 (100.0%)	0.202
Once a semester	5 (55.6%)	4 (44.4%)		2 (50.0%)	2 (50.0%)		3 (60.0%)	2 (40.0%)	
Once a month	49 (65.3%)	26 (34.7%)		43 (68.3%)	20 (31.7%)		6 (50.0%)	6 (50.0%)	
Once a fortnight	256 (64.0%)	144 (36.0%)		145 (65.6%)	76 (34.4%)		111 (62.0%)	68 (38.0%)	
Once a week	44 (66.7%)	22 (33.3%)		11 (57.9%)	8 (42.1%)		33 (70.2%)	14 (29.8%)	

*Adjust the height of desks and chairs*, *n* (%)									
Never	88 (66.7%)	44 (33.3%)	0.008	37 (61.7%)	23 (38.3%)	0.629	51 (70.8%)	21 (29.2%)	<0.001
Once a year	44 (48.4%)	47 (51.6%)		19 (70.4%)	8 (29.8%)		25 (39.1%)	39 (60.9%)	
Once a semester	176 (65.9%)	91 (34.1%)		104 (63.0%)	61 (37.0%)		72 (70.6%)	30 (29.4%)	
Once every 2 to 3 months	46 (70.8%)	19 (29.2%)		41 (70.7%)	17 (29.3%)		5 (71.4%)	2 (28.6%)	

*Activity place during recess*, *n* (%)									
Teaching building	264 (63.9%)	149 (36.1%)	0.908	153 (66.5%)	77 (33.5%)	0.293	111 (60.7%)	72 (39.3%)	0.319
Outside teaching building	90 (63.4%)	52 (36.6%)		48 (60.0%)	32 (40.0%)		42 (67.7%)	20 (32.3%)	

*Time for homework per day*, *n* (%)									
<1 h	52 (65.0%)	28 (35.0%)	0.828	48 (69.6%)	21 (30.4%)	0.196	4 (36.4%)	7 (63.6%)	0.280
1–1.99 h	157 (63.1%)	92 (36.9%)		88 (66.2%)	45 (33.8%)		69 (59.5%)	47 (40.5%)	
2–2.99 h	106 (65.4%)	56 (34.6%)		47 (63.5%)	27 (36.5%)		59 (67.0%)	29 (33.0%)	
≥3 h	37 (62.7%)	22 (37.2%)		18 (56.3%)	14 (43.8%)		19 (70.4%)	8 (29.6%)	
Uncertain	2 (40.0%)	3 (60.0%)		0 (0.0%)	2 (100.0%)		2 (66.7%)	1 (33.3%)	

*Time for interest classes per week*, *n* (%)									
0 h	57 (60.0%)	38 (40.0%)	0.862	33 (67.3%)	16 (32.7%)	0.789	24 (52.2%)	22 (47.8%)	0.665
<1 h	17 (60.7%)	11 (39.3%)		14 (58.3%)	10 (41.7%)		3 (75.0%)	1 (25.0%)	
1–1.99 h	66 (64.7%)	36 (35.3%)		43 (67.2%)	21 (32.8%)		23 (60.5%)	15 (39.5%)	
2–2.99 h	81 (62.3%)	49 (37.7%)		37 (59.7%)	25 (40.3%)		44 (64.7%)	24 (35.3%)	
≥3 h	129 (66.2%)	66 (33.8%)		73 (66.4%)	37 (33.6%)		56 (65.9%)	29 (34.1%)	
Uncertain	4 (80.0%)	1 (20.0%)		1 (100.0%)	0 (0.0%)		3 (75.0%)	1 (25.0%)	

*Parents limit sports time for study*, *n* (%)									
Often	28 (60.9%)	18 (39.1%)	0.788	17 (65.4%)	9 (34.6%)	0.996	11 (55.0%)	9 (45.0%)	0.523
Sometimes	111 (65.7%)	58 (34.3%)		60 (64.5%)	33 (35.5%)		51 (67.1%)	25 (32.9%)	
Never	215 (63.2%)	125 (36.8%)		124 (64.9%)	67 (35.1%)		91 (61.1%)	58 (38.9%)	

*Parents limit electronic products*, *n* (%)									
Yes	323 (64.3%)	179 (35.7%)	0.399	182 (66.2%)	93 (33.8%)	0.165	141 (62.1%)	86 (37.9%)	0.701
No	31 (58.5%)	22 (41.5%)		19 (54.3%)	16 (45.7%)		12 (66.7%)	6 (33.3%)	

*Sit more than one-punch distance from the edge of the table when reading and writing*, *n* (%)									
Never	23 (59.0%)	16 (41.0%)	0.560	15 (57.7%)	11 (42.3%)	0.606	8 (61.5%)	5 (38.5%)	0.981
Sometimes	108 (62.1%)	66 (37.9%)		57 (62.6%)	34 (37.4%)		51 (61.4%)	32 (38.6%)	
Often	129 (62.9%)	76 (37.1%)		56 (63.6%)	32 (36.4%)		73 (62.4%)	44 (37.6%)	
Always	94 (68.6%)	43 (31.4%)		73 (69.5%)	32 (30.5%)		21 (65.6%)	11 (34.4%)	

*The distance between eyes and books is more than 33 cm when reading and writing*, *n* (%)									
Never	21 (53.8%)	18 (46.2%)	0.276	15 (57.7%)	11 (42.3%)	0.606	7 (50.0%)	7 (50.0%)	0.660
Sometimes	114 (62.3%)	69 (37.7%)		57 (62.6%)	34 (37.4%)		52 (66.7%)	26 (33.3%)	
Often	137 (63.4%)	79 (36.6%)		56 (63.6%)	32 (36.4%)		79 (61.2%)	50 (38.8%)	
Always	82 (70.1%)	35 (29.9%)		73 (69.5%)	32 (30.5%)		15 (62.5%)	9 (37.5%)	

*The distance between fingers and nib is about 3.3 cm when reading and writing*, *n* (%)									
Never	31 (53.4%)	27 (46.6%)	0.062	15 (53.6%)	13 (46.4%)	0.073	16 (53.3%)	14 (46.7%)	0.723
Sometimes	65 (61.9%)	40 (38.1%)		38 (61.3%)	24 (38.7%)		27 (62.8%)	16 (37.2%)	
Often	125 (61.3%)	79 (38.7%)		44 (57.9%)	32 (42.1%)		81 (63.3%)	47 (36.7%)	
Always	133 (70.7%)	55 (29.3%)		104 (72.2%)	40 (27.8%)		29 (65.9%)	15 (34.1%)	

*Teachers remind the gestures of reading and writing*, *n* (%)									
Never	28 (65.1%)	15 (34.9%)	0.224	18 (66.7%)	9 (33.3%)	0.906	10 (62.5%)	6 (37.5%)	0.143
Sometimes	75 (56.4%)	58 (43.6%)		46 (61.3%)	29 (38.7%)		29 (50.0%)	29 (50.0%)	
Often	90 (64.7%)	49 (35.3%)		47 (65.3%)	25 (34.7%)		43 (64.2%)	24 (35.8%)	
Always	161 (67.1%)	79 (32.9%)		90 (66.2%)	46 (33.8%)		71 (68.3%)	33 (31.7%)	

*Parents remind the gestures of reading and writing*, *n* (%)									
Never	13 (48.1%)	14 (51.9%)	0.145	9 (50.0%)	9 (50.0%)	0.379	4 (44.4%)	5 (55.6%)	0.462
Sometimes	60 (64.5%)	33 (35.5%)		37 (67.3%)	18 (32.7%)		23 (60.5%)	15 (39.5%)	
Often	108 (60.3%)	71 (39.7%)		55 (61.1%)	35 (38.9%)		53 (59.6%)	36 (40.4%)	
Always	173 (67.6%)	83 (32.4%)		100 (68.0%)	47 (32.0%)		73 (67.0%)	36 (33.0%)	

*Watching TV per day*, *n* (%)									
Never	51 (66.2%)	26 (33.8%)	0.085	33 (68.8%)	15 (31.3%)	0.051	18 (62.1%)	11 (37.9%)	0.494
<1 h	187 (61.5%)	117 (38.5%)		93 (61.6%)	58 (38.4%)		94 (61.4%)	59 (38.6%)	
1–1.99 h	86 (69.4%)	38 (30.6%)		56 (69.1%)	25 (30.9%)		30 (69.8%)	13 (30.2%)	
2–2.99 h	21 (72.4%)	8 (27.6%)		14 (87.5%)	2 (12.5%)		7 (53.8%)	6 (46.2%)	
3–3.99 h	3 (75.0%)	1 (25.0%)		1 (50.0%)	1 (50.0%)		2 (100.0%)	0 (0.0%)	
≥4 h	6 (35.3%)	11 (64.7%)		4 (33.3%)	8 (66.7%)		2 (40.0%)	3 (60.0%)	

*Using computers per day*, *n* (%)									
Never	190 (64.0%)	107 (36.0%)	0.250	122 (66.7%)	61 (33.3%)	0.221	68 (59.6%)	46 (40.4%)	0.637
<1 h	138 (65.4%)	73 (34.6%)		57 (65.5%)	30 (34.5%)		81 (65.3%)	43 (34.7%)	
1–1.99 h	21 (52.5%)	19 (47.5%)		17 (51.5%)	16 (48.5%)		4 (57.1%)	3 (42.9%)	
2–2.99 h	3 (100.0%)	0 (0.0%)		3 (100.0%)	0 (0.0%)		0	0	
3–3.99 h	2 (50.0%)	2 (50.0%)		0	0		0	0	
≥4 h				2 (50.0%)	2 (50.0%)		0	0	

*Using mobile devices more than 1 hour per day*, *n* (%)									
Yes	278 (63.3%)	161 (36.7%)	0.744	48 (63.2%)	28 (36.8%)	0.724	28 (68.3%)	13 (31.7%)	0.378
No	76 (65.0%)	41 (35.0%)		153 (65.4%)	81 (34.6%)		125 (61.0%)	80 (39.0%)	

*Reading books or electronic screens in direct sunlight*, *n* (%)									
Never	264 (62.3%)	160 (37.7%)	0.205	159 (64.9%)	86 (35.1%)	0.543	105 (58.7%)	74 (41.3%)	0.151
Sometimes	79 (68.7%)	36 (31.3%)		33 (63.5%)	19 (36.5%)		46 (73.0%)	17 (27.0%)	
Often	4 (50.0%)	4 (50.0%)		3 (50.0%)	3 (50.0%)		1 (50.0%)	1 (50.0%)	
Always	7 (87.5%)	1 (12.5%)		6 (85.7%)	1 (14.3%)		1 (100.0%)	0 (0.0%)	

*Turn off the light when looking at the electronic screen after dark*, *n* (%)									
Never	282 (63.5%)	162 (36.5%)	0.899	171 (65.0%)	92 (35.0%)	0.997	111 (61.3%)	70 (38.7%)	0.572
Sometimes	61 (66.3%)	31 (33.7%)		21 (63.6%)	12 (36.4%)		40 (67.8%)	19 (32.2%)	
Often	5 (55.6%)	4 (44.4%)		4 (66.7%)	2 (33.3%)		1 (33.3%)	2 (66.7%)	
Always	6 (60.0%)	4 (40.0%)		5 (62.5%)	3 (37.5%)		1 (50.0%)	1 (50.0%)	

*Reading or looking at electronic screens by lying*, *n* (%)									
Never	189 (64.3%)	105 (35.7%)	0.732	121 (65.4%)	64 (34.6%)	0.256	68 (62.4%)	41 (37.6%)	0.968
Sometimes	129 (62.9%)	76 (37.1%)		64 (64.0%)	36 (36.0%)		65 (61.9%)	40 (38.1%)	
Often	31 (62.0%)	19 (38.0%)		12 (57.1%)	9 (42.9%)		19 (65.5%)	10 (34.5%)	
Always	5 (83.3%)	1 (16.7%)		4 (100.0%)	0 (0.0%)		1 (50.0%)	1 (50.0%)	

*Reading or looking at electronic screens when walking or taking a bus*, *n* (%)									
Never	278 (63.2%)	162 (36.8%)	0.805	165 (65.5%)	87 (34.5%)	0.756	113 (60.1%)	75 (39.9%)	0.376
Sometimes	71 (65.7%)	37 (34.3%)		33 (61.1%)	21 (38.9%)		38 (70.4%)	16 (29.6%)	
Often	5 (71.4%)	2 (28.6%)		3 (75.0%)	1 (25.0%)		2 (66.7%)	1 (33.3%)	
Always	0	0		0	0		0	0	

*The lamp used when reading after dark*, *n* (%)									
Both desk lamp and roof lamp	230 (65.2%)	123 (34.8%)	0.354	125 (64.8%)	68 (35.2%)	0.237	105 (65.6%)	55 (34.4%)	0.362
Only desk lamp	24 (53.3%)	21 (46.7%)		14 (50.0%)	14 (50.0%)		10 (58.8%)	7 (41.2%)	
Only roof lamp	99 (63.5%)	57 (36.5%)		61 (69.3%)	27 (30.7%)		38 (55.9%)	30 (44.1%)	
Others	1 (100.0%)	0 (0.0%)		1 (100.0%)	0 (0.0%)		0	0	

*The distance between eyes and screens more than 66 cm when using computers*, *n* (%)									
Never using computers	115 (68.9%)	52 (31.1%)	0.483	77 (73.3%)	28 (26.7%)	0.238	38 (61.3%)	24 (38.7%)	0.844
Never	34 (59.6%)	23 (40.4%)		12 (54.5%)	10 (45.5%)		22 (62.9%)	13 (37.1%)	
Sometimes	72 (62.6%)	43 (37.4%)		26 (63.4%)	15 (36.6%)		46 (62.2%)	28 (37.8%)	
Often	57 (64.8%)	31 (35.2%)		23 (60.5%)	15 (39.5%)		34 (68.0%)	16 (32.0%)	
Always	76 (59.4%)	52 (40.6%)		63 (60.6%)	41 (39.4%)		13 (54.2%)	11 (45.8%)	

*The distance between eyes and TV more than 3 m when watching TV*, *n* (%)									
Never watching TV	28 (65.1%)	15 (34.9%)	0.319	17 (70.8%)	7 (29.2%)	0.318	11 (57.9%)	8 (42.1%)	0.123
Never	27 (62.8%)	16 (37.2%)		13 (52.0%)	12 (48.0%)		14 (77.8%)	4 (22.2%)	
Sometimes	101 (70.6%)	42 (29.4%)		39 (70.9%)	16 (29.1%)		62 (70.5%)	26 (29.5%)	
Often	72 (63.2%)	42 (36.8%)		45 (70.3%)	19 (29.7%)		27 (54.0%)	23 (46.0%)	
Always	126 (59.4%)	86 (40.6%)		87 (61.3%)	55 (38.7%)		39 (55.7%)	31 (44.3%)	

*Time on outdoor activities at daytime per day*, *n* (%)									
<1 h	60 (59.4%)	41 (40.6%)	0.130	39 (62.9%)	23 (37.1%)	0.276	21 (53.8%)	18 (46.2%)	0.343
1–1.99 h	184 (68.9%)	83 (31.1%)		110 (69.2%)	49 (30.8%)		74 (68.5%)	34 (31.5%)	
2–2.99 h	61 (62.2%)	37 (37.8%)		23 (67.6%)	11 (32.4%)		38 (59.4%)	26 (40.6%)	
≥3 h	40 (54.1%)	34 (45.9%)		24 (53.3%)	21 (46.7%)		16 (55.2%)	13 (44.8%)	
Uncertain	9 (60.0%)	6 (40.0%)		5 (50.0%)	5 (50.0%)		4 (80.0%)	1 (20.0%)	

*Sleeping time more than 8 h*, *n* (%)									
Yes	346 (64.6%)	190 (35.4%)	0.025	197 (66.1%)	101 (33.9%)	0.043	149 (62.6%)	89 (37.4%)	0.724
No	8 (40.0%)	12 (60.0%)		4 (33.3%)	8 (66.7%)		4 (50.0%)	4 (50.0%)	

*Father or mother has myopia*, *n* (%)									
Yes	222 (69.4%)	98 (30.6%)	0.001	120 (70.6%)	50 (29.4%)	0.019	102 (68.0%)	48 (32.0%)	0.024
No	132 (56.2%)	103 (43.8%)		81 (57.9%)	59 (42.1%)		51 (53.7%)	44 (46.3%)	

*Performed the examination of myopia in the past year*, *n* (%)									
Yes	336 (64.2%)	187 (35.8%)	0.361	189 (65.4%)	100 (34.6%)	0.444	147 (62.8%)	87 (37.2%)	0.814
No	18 (56.3%)	14 (43.8%)		12 (57.1%)	9 (42.9%)		6 (54.5%)	5 (45.5%)	

**Table 3:** The associations between factors and myopia using multivariate logistic regression.

Parameters	All (*n* = 556)		School A (*n* = 310)		School B (*n* = 246)	
	OR (95% CI)	*P* value	OR (95% CI)	*P* value	OR (95% CI)	*P* value
*Age*	1.57 (0.87, 2.80)	0.131	1.47 (0.55, 3.92)	0.437	1.75 (0.82, 3.75)	0.149

*Grade*						
4	0.94 (0.26, 3.37)	0.919	1.20 (0.15, 9.91)	0.866	0.97 (0.16, 6.08)	0.976
5	1.03 (0.48, 2.21)	0.936	0.88 (0.27, 2.91)	0.837	1.28 (0.39, 4.16)	0.686
6	Reference		Reference		Reference	

*Gender*						
Male	0.98 (0.68, 1.42)	0.914	0.87 (0.53, 1.44)	0.596	1.08 (0.60, 1.95)	0.795
Female	Reference		Reference		Reference	

*Change class seats*						
Never	—	—	—	—	—	
Once a semester	1.09 (0.25, 4.82)	0.907	0.83 (0.09, 7.82)	0.867	1.07 (0.13, 8.73)	0.950
Once a month	1.08 (0.52, 2.26)	0.829	1.17 (0.38, 3.64)	0.781	0.58 (0.12, 2.75)	0.497
Once a fortnight	1.10 (0.61, 2.01)	0.748	1.23 (0.43, 3.52)	0.694	1.15 (0.50, 2.64)	0.749
Once a week	Reference		Reference		Reference	

*Adjust the height of desks and chairs*						
Never	Reference		Reference		Reference	
Once a year	0.37 (0.21, 0.67)	0.001	1.07 (0.36, 3.13)	0.908	0.26 (0.11, 0.62)	0.003
Once a semester	0.60 (0.35, 0.97)	0.037	0.74 (0.34, 1.60)	0.450	0.55 (0.22, 1.34)	0.188
Once every 2 to 3 months	0.76 (0.37, 1.56)	0.452	0.98 (0.38, 2.52)	0.973	0.82 (0.13, 5.13)	0.827

*Sleeping time more than 8 h*						
Yes	Reference		Reference		Reference	
No	0.45 (0.17, 1.18)	0.103	0.29 (0.08, 1.03)	0.055	1.15 (0.21, 6.18)	0.870

*Father or mother has myopia*						
Yes	Reference		Reference		Reference	
No	0.51 (0.35, 0.74)	<0.001	0.56 (0.34, 0.93)	0.026	0.45 (0.25, 0.83)	0.009

## Data Availability

The data used to support the findings of this study are presented in the tables.

## Abbreviations

AL: Axial length
CR: Corneal radius
UCVA: Uncorrected visual acuity
OR: Odds ratio
CIs: Confidence intervals.
